# Effects of Irregular Feeding on the Daily Fluctuations in mRNA Expression of the Neurosecretory Protein GL and Neurosecretory Protein GM Genes in the Mouse Hypothalamus

**DOI:** 10.3390/ijms22042109

**Published:** 2021-02-20

**Authors:** Atsuki Kadota, Eiko Iwakoshi-Ukena, Keisuke Fukumura, Kenshiro Shikano, Yuki Narimatsu, Megumi Furumitsu, Kazuyoshi Ukena

**Affiliations:** 1 Laboratory of Neurometabolism, Graduate School of Integrated Sciences for Life, Hiroshima University, Higashi-Hiroshima, Hiroshima 739-8521, Japan; m193645@hiroshima-u.ac.jp (A.K.); iwakoshi@hiroshima-u.ac.jp (E.I.-U.); kfuku@hiroshima-u.ac.jp (K.F.); kshikano@oita-u.ac.jp (K.S.); m192783@hiroshima-u.ac.jp (Y.N.); mfurumi@hiroshima-u.ac.jp (M.F.); 2 Department of Neurophysiology, Faculty of Medicine, Oita University, Yufu, Oita 879-5593, Japan

**Keywords:** neurosecretory protein GL, neurosecretory protein GM, rhythmicity, food intake, fat accumulation, environmental light/dark cycle, feeding restriction

## Abstract

Circadian desynchrony induced by a long period of irregular feeding leads to metabolic diseases, such as obesity and diabetes mellitus. The recently identified neurosecretory protein GL (NPGL) and neurosecretory protein GM (NPGM) are hypothalamic small proteins that stimulate food intake and fat accumulation in several animals. To clarify the mechanisms that evoke feeding behavior and induce energy metabolism at the appropriate times in accordance with a circadian rhythm, diurnal fluctuations in *Npgl* and *Npgm* mRNA expression were investigated in mice. Quantitative RT-PCR analysis revealed that the mRNAs of these two genes were highly expressed in the mediobasal hypothalamus during the active dark phase under ad libitum feeding. In mice restricted to 3 h of feeding during the inactive light phase, the *Npgl* mRNA level was augmented in the moment prior to the feeding period and the midnight peak of *Npgm* mRNA was attenuated. Moreover, the mRNA expression levels of clock genes, feeding regulatory neuropeptides, and lipid metabolic enzymes in the central and peripheral tissues were comparable to those of central *Npgl* and *Npgm*. These data suggest that *Npgl* and *Npgm* transcription fluctuates daily and likely mediates feeding behavior and/or energy metabolism at an appropriate time according to the meal timing.

## 1. Introduction

In many industrialized countries, the health of shift workers has become an issue of concern, given that night shift and rotating shift work are associated with an increased prevalence of various disorders, such as diabetes, cardiovascular disease, and cancer [1,2]. The body’s biological clock regulates the sleep/wake cycle, body temperature, metabolism, and hormone secretion, allowing the physiological processes to adapt to the light/dark cycle [3]. In mammals, these circadian rhythms are controlled through the suprachiasmatic nucleus (SCN), the central region in the hypothalamus that generates neuronal and hormonal activity [4,5,6]. Endogenous circadian oscillation is based on transcriptional feedback circuits regulated by core clock genes, such as *Clock*, brain and muscle ARNT-like 1 (*Bmal1*), period (*Per1*, *Per2*, and *Per3*), and cryptochrome (*Cry1* and *Cry2*) [7,8,9]. CLOCK and BMAL1 heterodimerize and inhibit their own expression by inducing the transcription of their transcriptional repressors, the *Per* and *Cry* genes [10,11,12]. The CLOCK and BMAL1 complexation also stimulates the rhythmic transcription of the gene coding for nuclear receptor subfamily 1 group D member 1 (*Nr1d1*), an orphan nuclear receptor known as REB-ERBα, which then feeds back to repress the transcription of *Bmal1* [13,14]. The daily biological rhythm generated by these clock genes is adjusted to an approximately 24 h cycle. It is also known that peripheral tissues, such as liver, skeletal muscle, and adipose tissue, have independent biological rhythms in accordance with the light/dark cycle [7,15]. These rhythms are modulated by the autonomic nervous system and endocrine system through output signals from the SCN [3].

The biological clocks are strongly synchronized not only by the light/dark cycle but also by feeding [16,17]. Feeding at the wrong time, such as eating during the inactive phase, easily changes the expression patterns of clock genes in the peripheral tissues [18,19]. Thus, harmonization between the light/dark cycle and the feeding time is important for maintaining an appropriate biological rhythm for energy homeostasis. It has been reported that hypothalamic orexigenic/anorexigenic neuropeptides (also known as feeding regulatory neuropeptides), such as neuropeptide Y (NPY), orexin (ORX), melanin-concentrating hormone (MCH), and proopiomelanocortin (POMC), show rhythmic activity under ad libitum feeding (ALF) [20,21,22]. By contrast, the daily rhythm of neuronal activity or secretion following the light/dark cycle in rats is disrupted by feeding during the daytime, the inactive phase for rodents [22,23]. Therefore, feeding during an irregular period can disturb the function of the SCN, eventually inducing circadian desynchrony and leading to metabolic diseases, such as obesity and type 2 diabetes [24,25,26]. In addition to neuropeptides, lipogenic genes, such as the gene encoding fatty acid synthase (*Fasn*), display daily transcription rhythms that can also be easily influenced by the wrong time feeding in peripheral tissues [27,28]. Thus, the daily rhythms of energy metabolism are strongly associated with feeding to handle dietary nutrients appropriately. Although considerable studies on the circadian control of biological rhythms have been conducted as described above, the central and peripheral relationships between the activities of the neuropeptides and the daily rhythms of feeding and energy metabolism are not fully understood.

Recently, we discovered two novel cDNAs (viz., neurosecretory protein GL (*Npgl*) and its paralog neurosecretory protein GM (*Npgm*)) that encode the precursors of NPGL and NPGM, respectively, in chicken, rat, mouse, and human brains [29,30,31,32]. We revealed that NPGL is expressed in the mediobasal hypothalamus (MBH) of mouse and rat brains and promotes feeding and fat accumulation [30,31,33]. It is likely that NPGM has a similar biological function because both neurosecretory proteins are produced in the same neuron [32]. However, whether NPGL and NPGM are involved in the biological rhythm of feeding behavior and energy metabolism has not been clarified. Therefore, the principal aim of this study was to reveal whether the mRNA expression levels of *Npgl* and *Npgm* display daily rhythms in the MBH of mice. The daily mRNA expression profiles of these two genes were investigated under ALF and time-restricted feeding (RF) conditions to investigate the environmental light/dark cycle and feeding time effects. Additionally, the circadian expression of the representative clock genes (*Bmal1*, *Per2*, *Cry1*, and *Nr1d1*) as described above was confirmed in several tissues. The diurnal mRNA expression profiles of the crucial feeding regulatory neuropeptides and precursor protein (*Npy*, *Orx*, *Mch*, and *Pomc*) as mentioned above and lipid metabolic enzymes (typical lipogenic and lipolytic enzymes) including *Fasn* in central and peripheral tissues were also analyzed because NPGL might regulate feeding and promote de novo lipogenesis [30,31,33,34,35].

## 2. Results

### 2.1. Daily mRNA Expression Profiles of Clock Genes in Central and Peripheral Tissues and Effects of the Feeding Times

**Figure 1:**
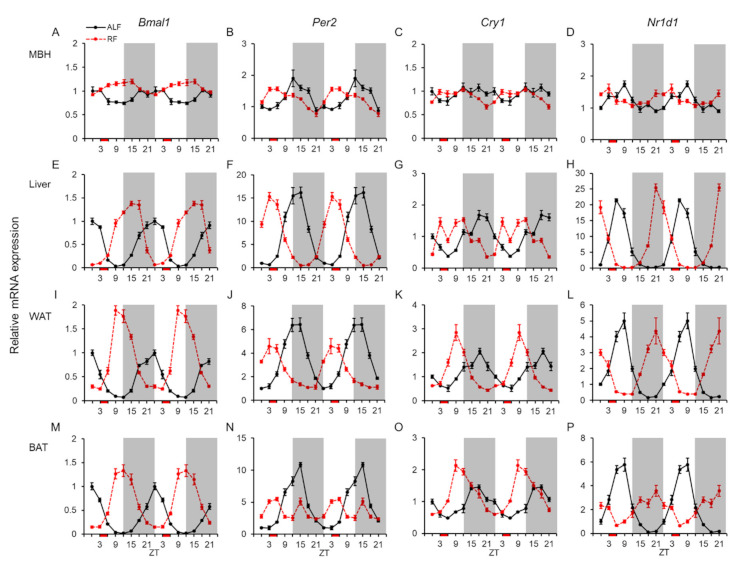
mRNA expression levels of clock genes in the central and peripheral tissues. Double-plotted graphs showing the mRNA expression levels of the brain and muscle ARNT-like 1 (*Bmal1*), period 2 (*Per2*), cryptochrome 1 (*Cry1*), and nuclear receptor subfamily 1 group D member 1 (*Nr1d1*) genes in the mediobasal hypothalamus (MBH: **A**–**D**), liver (**E**–**H**), white adipose tissue (WAT: **I**–**L**), and brown adipose tissue (BAT: **M**–**P**) under ad libitum feeding (ALF: Black lines) and time-restricted feeding (RF: Red lines) schedules. Data are expressed as the mean ± standard error of the mean, *n* = 5–6 mice per datum point.

We investigated the mRNA expression profiles of four clock genes (*Bmal1*, *Per2*, *Cry1*, and *Nr1d1*) to examine their rhythmicity and responses to feeding times. The mRNA expression levels were measured in the MBH, liver, white adipose tissue (WAT), and brown adipose tissue (BAT) of mice under ALF and RF (i.e., only 3 h during the inactive light phase) (Figure 1).

Each clock gene showed similar mRNA expression peaks of daily changes in the four different tissues (Figure 1). Statistical analysis using one-way analysis of variance (ANOVA) showed that the expression of these clock genes fluctuated daily in the MBH, liver, WAT, and BAT under ALF, except for that of *Cry1* in the MBH (Table 1). Moreover, the JTK-Cycle algorithm, which validates 24 h rhythms of clock gene expression, revealed that all four genes displayed rhythmicities of mRNA expression in all the examined tissues under ALF (Table 2). Additionally, the amplitudes of fluctuation for the four clock genes were lower in the MBH than in the peripheral tissues (Figure 1 and Table 2).

By contrast, the mRNA expression of each clock gene under RF displayed obvious daily fluctuations in each tissue (Table 1). Furthermore, a high level of *Cry1* mRNA expression was observed in the liver at zeitgeber time (ZT) 3, just before refeeding, under RF (Figure 1G). Rhythmicities of mRNA expression under RF were also demonstrated for all the clock genes, except for *Per2* in BAT, which displayed a peak at ZT 15 as well as that under ALF (Figure 1N and Table 2). Additionally, the amplitudes of *Bmal1* in WAT and *Cry1* in WAT and BAT under RF were larger than those under ALF, while the amplitudes of *Per2* in WAT and BAT and *Nr1d1* in BAT under RF were smaller than those under ALF (Table 2). The acrophases for the four clock genes under RF were moved forward by approximately 9 h relative to those under ALF in the four different tissues (Table 2).

Statistical analysis using two-way ANOVA, which shows a difference between ALF and RF, indicated significant effects of feeding for *Bmal1* in the four different tissues, *Per2* in WAT and BAT, *Cry1* in BAT, and *Nr1d1* in liver (Table 3). Additionally, it showed significant effects of time and interaction of feeding × time in the four different tissues, except for time of *Bmal1* in the MBH (Table 3).

### 2.2. Daily mRNA Expression Profiles of Npgl and Npgm in MBH and Effects of the Feeding Times

**Figure 2:**
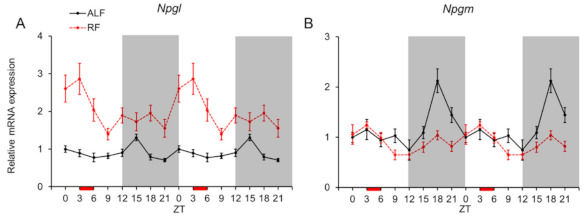
mRNA expression levels of the neurosecretory protein GL (*Npgl*) and neurosecretory protein GM (*Npgm*) genes in the mediobasal hypothalamus (MBH). Double-plotted graphs showing the mRNA expression levels of *Npgl* (**A**) and *Npgm* (**B**) under ad libitum feeding (ALF: Black lines) and time-restricted feeding (RF: Red lines) schedules. Data are expressed as the mean ± standard error of the mean, *n* = 5–6 mice per datum point.

Next, we analyzed the diurnal mRNA expression profiles of *Npgl* and *Npgm* (both known to be related to feeding and fat accumulation) in the MBH under ALF and RF (Figure 2). Statistical analysis using one-way ANOVA revealed that the levels of *Npgl* and *Npgm* expression fluctuated daily under ALF (Figure 2 and Table 1), while the JTK-Cycle did not confirm the rhythmicity for *Npgl* neither for ALF nor for RF group. The highest *Npgl* expression level was at ZT 15 (vs. ZT 6, 9, 18, and 21, *P* < 0.05 by the post-hoc Tukey’s multiple comparisons test; Figure 2A), whereas the peak of *Npgm* mRNA expression was at ZT 18 (vs. ZT 0, 3, 6, 9, 12, and 15, *P* < 0.05 by the post-hoc Tukey’s multiple comparisons test; Figure 2B). The JTK-Cycle analysis showed that the acrophase for *Npgm* was at ZT 19.5 (Table 2). 

The mRNA expression of *Npgl* under RF was upregulated at ZT 3, just before refeeding (vs. ZT 9, *p* = 0.031 by the post-hoc Tukey’s multiple comparisons test; Figure 2A). Additionally, a peak of *Npgm* expression at ZT 18 under RF was reduced compared with that of ALF (Figure 2B). Daily fluctuations in *Npgl* and *Npgm* mRNA expression were also observed under RF (Figure 2 and Table 1). Statistical analysis using two-way ANOVA indicated significant effects of feeding, time, and interaction of feeding × time for *Npgl* and *Npgm* (Table 3).

### 2.3. Daily mRNA Expression Profiles of Orexigenic and Anorexigenic Genes in MBH and Effects of the Feeding Times

**Figure 3:**
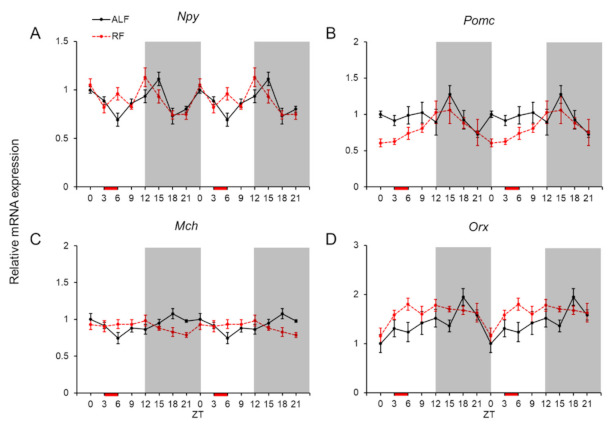
mRNA expression levels of orexigenic and anorexigenic genes in the mediobasal hypothalamus (MBH). Double-plotted graphs showing the mRNA expression levels of the neuropeptide Y (*Npy*: **A**), proopiomelanocortin (*Pomc*: **B**), melanin-concentrating hormone (*Mch*: **C**), and orexin (*Orx*: **D**) genes under ad libitum feeding (ALF: Black lines) and time-restricted feeding (RF: Red lines) schedules. Data are expressed as the mean ± standard error of the mean, *n* = 4–6 mice per datum point.

The mRNA expression levels of representative orexigenic and anorexigenic genes (viz., *Npy*, *Pomc*, *Mch*, and *Orx*) were measured because the function of NPGL in feeding could be exerted via these neuropeptides (Figure 3). Only *Npy* and *Mch* showed daily fluctuations under ALF, although a high expression level of all four genes tended to be observed at ZT 15 or ZT 18 during the dark phase (Figure 3 and Table 1). Only *Mch* showed a significant rhythmicity in its mRNA expression under ALF (Table 2). Under RF, there was hardly any phase shifting caused by the feeding time for any of the genes (Figure 3). The mRNA expression level of *Npy* fluctuated daily under RF, and only *Pomc* displayed a significant rhythmicity (Figure 3 and Table 1 and Table 2). Statistical analysis using two-way ANOVA indicated no effects of feeding and significant effects of time and interaction of feeding × time for *Npy* and *Orx*, and a significant effect of interaction of feeding × time for *Mch* (Table 3).

### 2.4. Daily mRNA Expression Profiles of Genes Related to Lipid Metabolism in Peripheral Tissues and Effects of the Feeding Times

**Figure 4:**
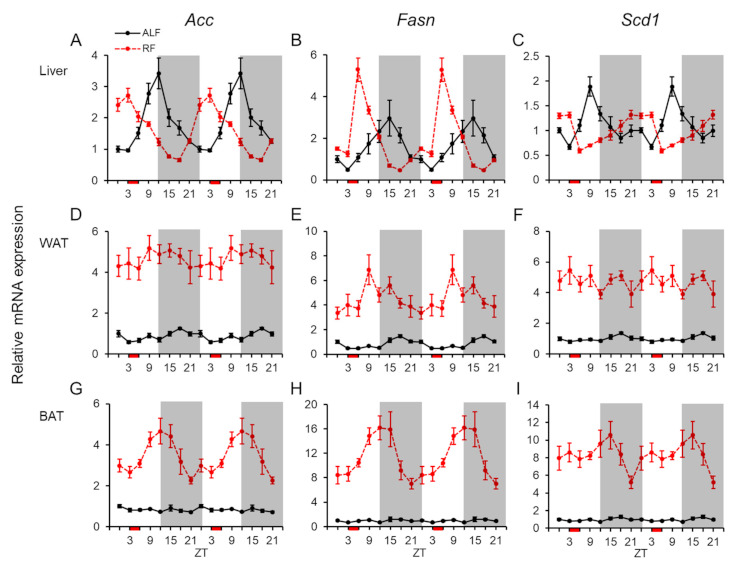
mRNA expression levels of lipogenic genes in the peripheral tissues. Double-plotted graphs showing the mRNA expression levels of the acetyl-CoA carboxylase (*Acc*), fatty acid synthase (*Fasn*), and stearoyl-CoA desaturase 1 (*Scd1*) genes in the liver (**A–C**), white adipose tissue (WAT: **D–F**), and brown adipose tissue (BAT: **G–I**) under ad libitum feeding (ALF: Black lines) and time-restricted feeding (RF: Red lines) schedules. Data are expressed as the mean ± standard error of the mean, *n* = 5–6 mice per datum point.

The mRNA expression profiles of the lipogenic genes (viz., acetyl-CoA carboxylase (*Acc*), *Fasn*, and stearoyl-CoA desaturase 1 (*Scd1*)) were investigated because NPGL facilitates fat accumulation in adipose tissue. The expression levels of these lipogenic genes were measured in the liver, WAT, and BAT under ALF and RF (Figure 4). In the liver, all three genes displayed clear daily fluctuations and rhythmicities in their mRNA expression under ALF and RF (Figure 4A–C and Table 1 and Table 2). Under RF, the acrophases were shifted forward by 7.5–12 h relative to those under ALF (Table 2). Statistical analysis using two-way ANOVA indicated no effects of feeding and significant effects of time and interaction of feeding × time for all three genes in the liver (Table 3). In WAT, the mRNA expressions of all three lipogenic genes were upregulated under RF compared with those under ALF (Figure 4). The mRNA expression levels of *Acc* and *Fasn* exhibited daily fluctuations and rhythmicities under ALF but not under RF, while the *Scd1* mRNA expression fluctuated daily under ALF (Figure 4D–F and Table 1 and Table 2). Statistical analysis using two-way ANOVA indicated significant effects of feeding and no effects of time and interaction of feeding × time for *Acc*, *Fasn*, and *Scd1*, except for interaction of feeding × time for *Fasn* in WAT (Table 3). By contrast, in BAT, the mRNA expressions of three lipogenic genes were highly upregulated under RF, likely in WAT (Figure 4). The *Acc* and *Fasn* expressions displayed daily fluctuations and rhythmicities under RF but not under ALF in BAT (Figure 4G, H and Table 1 and Table 2). Statistical analysis using two-way ANOVA indicated significant effects of feeding, time, and interaction of feeding × time for *Acc* and *Fasn*, and a significant effect of feeding and no effects of time and interaction of feeding × time for *Scd1* in BAT (Table 3).

**Figure 5:**
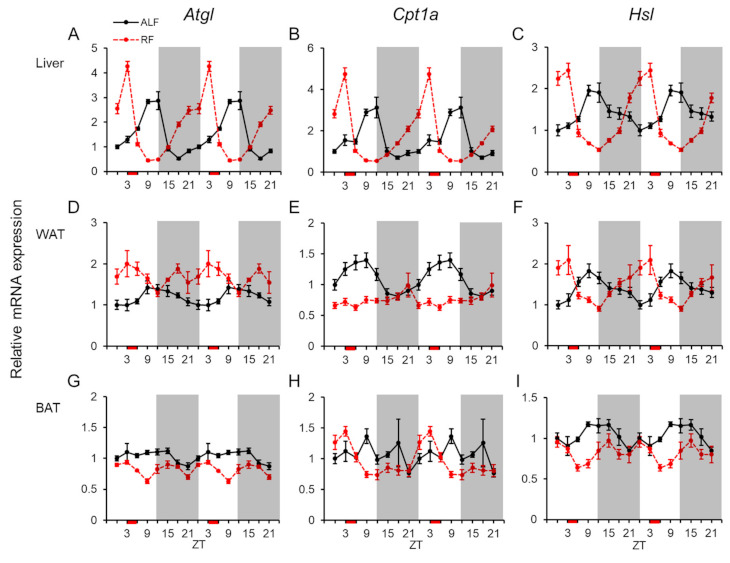
mRNA expression levels of lipolytic genes in the peripheral tissues. Double-plotted graphs showing the mRNA expression levels of the adipose triglyceride lipase (*Atgl*), carnitine palmitoyl transferase 1a (*Cpt1a*), and hormone-sensitive lipase (*Hsl*) genes in the liver (**A**–**C**), white adipose tissue (WAT: **D**–**F**), and brown adipose tissue (BAT: **G**–**I**) under ad libitum feeding (ALF: Black lines) and time-restricted feeding (RF: Red lines) schedules. Data are expressed as the mean ± standard error of the mean, *n* = 5–6 mice per datum point.

We also analyzed the mRNA expression profiles of the lipolytic genes (viz., adipose triglyceride lipase (*Atgl*), carnitine palmitoyl transferase 1a (*Cpt1a*), and hormone-sensitive lipase (*Hsl*)) under ALF and RF (Figure 5). In the liver, the mRNA expression levels of these genes displayed daily fluctuations and rhythmicities under both feeding conditions (Figure 5A–C and Table 1 and Table 2). The acrophases for three genes under RF were moved forward by 9 or 12 h compared with those under ALF (Table 2). Statistical analysis using two-way ANOVA indicated significant effects of feeding, time, and interaction of feeding × time for *Atgl*, and no effects of feeding and significant effects of time, and interaction of feeding × time for *Cpt1a* and *Hsl* in the liver (Table 3). In WAT, the mRNA expression levels of *Cpt1a* and *Hsl* fluctuated daily under ALF, whereas only *Hsl* showed a daily fluctuation under RF (Figure 5D–F and Table 1). Additionally, all the lipolytic genes displayed rhythmicities in their mRNA expression, except for *Cpt1a* under RF (Figure 5D–F and Table 2). Statistical analysis using two-way ANOVA indicated significant effects of feeding and interaction of feeding × time for *Atgl*, significant effects of feeding, time, and interaction of feeding × time for *Cpt1a*, and a significant effect of interaction of feeding × time for *Hsl* in WAT (Table 3). In BAT, the expression levels of *Atgl* and *Cpt1a* fluctuated daily under RF, whereas that of *Hsl* only fluctuated under ALF (Figure 5G–I and Table 1). Moreover, all the genes displayed rhythmicities under ALF in this tissue (Figure 5G–I and Table 2), whereas only *Cpt1a* had a rhythmicity under RF (Figure 5G–I and Table 2). Statistical analysis using two-way ANOVA indicated significant effects of feeding for *Atgl* and *Hsl*, and significant effects of time and interaction of feeding × time for all three genes in BAT (Table 3).

### 2.5. Daily Profiles of Blood Serum Glucose and Insulin Levels and Effects of the Feeding Times

Because the mRNA expression of *Npgl* has been suggested to be influenced by fasting or insulin in rodents [30,31], we also measured the diurnal blood serum levels of glucose and insulin in the mice (Figure S1). Similar to the daily fluctuations in *Npgl* and *Npgm* expression, the serum glucose and insulin levels fluctuated within a 24 h period (Table S1). The serum glucose level was high at ZT 6 under ALF, whereas it was low at ZT 3 under RF (Figure S1). Moreover, the serum insulin level was high at ZT 15 under ALF, whereas it was high at ZT 6, just after eating, under RF (Figure S1). 

## 3. Discussion

The daily light/dark cycle is an environmental factor that has a high influence on living organisms, especially on their circadian rhythms. A disruption of the circadian rhythm can cause neurodegenerative and psychiatric diseases [36,37]. Recently, it has been demonstrated that the feeding time and type of diets, such as a high-fat diet, could affect the circadian clock [27,38]. The feeding regulatory neuropeptides and lipid metabolic enzymes have been shown to display rhythmic activity, contributing to feeding and metabolic rhythms [20,21,22,27,28]. We had recently identified novel brain substances (NPGL and NPGM) in birds and rodents and demonstrated their important roles in regulating feeding behavior and fat accumulation [30,31,33,34,35]. However, the relationship between the biological rhythm and NPGL/NPGM expression has not been clarified. In the present study, we examined the daily mRNA expression profiles of *Npgl* and *Npgm* under ALF and RF with a 12 h light:12 h dark cycle (LD) in mice. Our data showed that the mRNA expression of these two genes fluctuated daily under ALF and that such fluctuations were altered by RF. The overall mRNA expression levels of *Npgl* were upregulated by RF. Compared with ALF, the amplitude and acrophase for *Npgm* mRNA under RF were smaller and moved forward by 18 h, respectively, due to a decrease in the highest peak at ZT 18 under ALF. We also found that mRNA expressions of many genes investigated in this study fluctuate daily and are influenced by RF in the central and peripheral tissues.

In this study, we confirmed the rhythmicity of mRNA expression of the clock genes (*Bmal1*, *Per2*, *Cry1*, and *Nr1d1*), similar to the results reported in previous studies [38,39]. The expression patterns of clock genes in the MBH did not correspond with those of *Npgl* and *Npgm*. Additionally, the JTK-Cycle analysis showed the rhythmicity in *Npgm* mRNA expression under ALF and RF. In the present study, we investigated the daily fluctuations of mRNA expressions under LD cycle in order to elucidate the influence of irregular feeding on *Npgl*/*Npgm* mRNA expressions. Further research under constant darkness is needed to reveal the effect of the biological clock on gene expressions. In addition, future studies are necessary to investigate whether these clock genes affect the transcription of *Npgm*, directly or indirectly. As the study of the promoter region of *Npgm* (also *Npgl*) has not been demonstrated yet, whether these regions possess the clock gene response elements such as E-box and retinoic acid-related orphan receptor response elements (ROREs) [11,13] is unknown.

The mRNA expression levels of *Npgl* and *Npgm* fluctuated daily under ALF, being the highest in the dark period; that is, at ZT 15 and ZT 18, respectively. This timing of expression may correspond to the effects of NPGL on feeding and fat deposition, because rodents have been shown to take in food and accumulate fats during the dark period [30,33]. Similar to *Npgl* and *Npgm*, *Npy* was the only gene that showed a daily fluctuation in its mRNA expression level under both ALF and RF. It is known that fasting induces *Npy* mRNA expression via AMP-activated protein kinase (AMPK), a cellular energy sensor [40,41]. AMPK activity displays circadian rhythms and can regulate the clock genes [42]. We had previously reported that *Npgl* mRNA was highly expressed in the fasting state, similar to *Npy* mRNA [30]. Therefore, future studies on the relationship between NPGL and energy sensors such as AMPK may contribute to our understanding of the mechanisms that regulate the daily fluctuations of *Npgl/Npgm* gene expression.

Our previous study suggested that NPGL was responsible to insulin and that it acted to maintain steady-state fat levels in concert with this hormone [30]. In this study, we found that the *Npgl* mRNA and serum insulin levels both peaked at ZT 15 under ALF. By contrast, it has been reported that the neuropeptides NPY, MCH, and ORX, and the precursor protein POMC show adaptation to the timing of feeding at the secretion or neuronal activity levels [22,23]. However, it has also been reported that daily changes in the mRNA levels of these neuropeptides are difficult to detect [43]. In this study, the mRNA expression levels of those genes showed slight changes under ALF and RF, similar to the data reported in the previous study [43], although *Pomc* under RF and *Mch* under ALF displayed rhythmicities. Hence, the apparent changes in the *Npgl* and *Npgm* mRNA levels under ALF and RF were characteristic features. 

It has been reported that rodents display food anticipatory activity (FAA), in that they looked for food in advance when their feeding time was restricted for several days [44,45]. We found high expression of *Npgl* at ZT 3, just before refeeding, under RF. Therefore, NPGL may be related to FAA. It has been suggested that the FAA center of rats is located in the dorsomedial hypothalamus (DMH) of the MBH [46,47]. Indeed, NPGL-immunoreactive fibers have been found to be distributed around the DMH in mice and rats [30,31]. Hence, it is possible that NPGL is involved in FAA. Therefore, our results suggest that *Npgl* mRNA expression might be more affected by the feeding time than by the light/dark cycle.

We also analyzed the daily mRNA expression profiles of lipid metabolic genes in the peripheral tissues and found that the changes were different among the various tissues. Although there are few reports about similar investigations, the mRNA expressions of lipid metabolic genes (*Acc*, *Fasn*, *Atgl*, and *Hsl*) showed similar diurnal expression patterns in the liver and WAT under ALF [27,28,38,48]. In the liver, the mRNAs of these lipid metabolic genes were highly expressed near the end of the light phase under ALF. Moreover, phase shifts in expression occurred under RF, similar to the clock genes and *Npgl*/*Npgm* genes. These results support that the changes might be induced by food-entrainable oscillator (FEO) [45]. In adipose tissues (WAT and BAT), the baselines of lipogenic enzyme (*Acc*, *Fasn*, and *Scd1*) were augmented by RF conditions. It has been reported that the cycle of starvation/refeeding increased the lipogenic enzyme mRNA expressions including *Acc* and *Fasn* in rats [49]. In this study, it is likely that RF might induce similar phenomena for fat accumulation in adipose tissues. In WAT, the mRNA expression levels of the lipogenic genes (*Acc* and *Fasn*) displayed similar daily fluctuations and rhythmicities to those of *Npgm* under ALF. We had recently reported that the intracerebroventricular infusion of NPGL and overexpression of *Npgl* in rodents affected the expression of lipid metabolic enzymes in WAT, suggesting that NPGL stimulates de novo lipogenesis [30,31,33,34,35]. Therefore, there may be some relationship in the daily fluctuations in expression levels between *Npgl*/*Npgm* and lipogenic enzymes in WAT under ALF. Contrary to those in WAT, the lipogenic genes (*Acc* and *Fasn*) in BAT showed rhythmicities in mRNA expression under RF but not under ALF. On the contrary, RF condition led to loss of rhythmicities in *Acc*, *Fasn*, and *Cpt1a* in WAT and *Atgl* and *Hsl* in BAT. These differences in reactivity to RF were likely caused by the difference in influence of the entrainer of each tissue. As described above, these rhythms are entrained through the autonomic nervous system, hormonal signals, and feeding [3]. Currently, the functional cascade of the MBH-produced NPGL/NPGM down through to the peripheral tissues is unknown. More studies on the downstream routes from NPGL/NPGM to the peripheral tissues, including WAT, are needed to elucidate the biological mechanisms of feeding and energy metabolism.

In summary, we revealed the daily fluctuations in both *Npgl* and *Npgm* mRNA expressions and the rhythmicity in *Npgm* mRNA expression in the mouse MBH for the first time. Importantly, these daily fluctuations could be altered by the timing of feeding. We also investigated the diurnal changes in mRNA expressions of feeding regulatory neuropeptides and lipid metabolic enzymes in addition to clock genes in the central and peripheral tissues under ALF and RF. The results suggested the presence of some relationship in mRNA expression levels between *Npgl*/*Npgm* and lipogenic enzymes in the liver and WAT under ALF. Taken together, the present data suggest that the production of endogenous NPGL/NPGM does fluctuate daily, and these neurosecretory proteins stimulate feeding behavior and/or energy metabolism at an appropriate time according to the meal timing. Future studies are needed in order to identify the daily fluctuations in the production of mature NPGL/NPGM at the translational level. Abnormal fluctuations in NPGL and NPGM caused by irregular feeding may be the cause of metabolic diseases, such as obesity. Therefore, NPGL and NPGM might be key factors for uncovering the mechanisms of circadian desynchrony and the ensuing metabolic abnormalities.

## 4. Materials and Methods

### 4.1. Animals

Male C57BL/6J mice (5 weeks old) were purchased from CLEA Japan (Tokyo, Japan) and entrained to a 12 h light:12 h dark cycle. The mice had ad libitum access to normal chow (CE-2; CLEA Japan) for 3 weeks before being randomly assigned to two feeding groups:

*ALF group*: Animals fed ad libitum, consuming most of their food during the dark phase.

*RF group*: Animals restricted to 3 h of food access during the light phase (i.e., from ZT 3 to ZT 6, where ZT 0 represents light on).

After 2 weeks on the designated feeding schedule, the animals were sacrificed and the MBH, liver, epididymal WAT, BAT, and blood serum were collected every 3 h over 24 h. The mRNA expression levels of the various genes were measured using the quantitative RT-PCR.

### 4.2. Quantitative RT-PCR

The MBH, liver, WAT, and BAT were dissected from the mice at ZT 0, 3, 6, 9, 12, 15, 18, and 21 and snap frozen in liquid nitrogen for RNA processing. The MBH was dissected out using fine forceps and small scissors from the bregma 0.00 mm (the optic chiasma) to the bregma −5.04 mm (the caudal border of the mammillary bodies) according to the Rat Brain Atlas of Paxinos and Watson [50]. These regions included the supraoptic nucleus, the DMH, the ventromedial hypothalamus, the arcuate nucleus, the lateral hypothalamic area, and the mammillary nucleus. Total RNA was extracted from the MBH and liver samples using TRIzol reagent (Life Technologies, Carlsbad, CA, USA) or from the WAT and BAT samples using QIAzol lysis reagent (Qiagen, Venlo, Netherlands) in accordance with the manufacturers’ instructions. First-strand cDNA was synthesized from total RNA using a ReverTra Ace kit (TOYOBO, Osaka, Japan). 

The primer sequences used in this study are listed in Table 4. PCR amplification was conducted with the THUNDERBIRD SYBR qPCR Mix (TOYOBO) using the following conditions: 95 °C for 20 s, followed by 40 cycles of 95 °C for 3 s and 60 °C for 30 s. The PCR products in each cycle were monitored using a Bio-Rad CFX Connect device (Bio-Rad Laboratories, Hercules, CA, USA). The relative quantification of expression of each gene was determined with the 2^−ΔΔCt^ method using the beta-actin (*Actb*) gene for MBH and liver and the ribosomal protein S18 (*Rps18*) gene for WAT and BAT as internal controls, respectively. The mRNA levels were expressed relative to the average value (set as 1) of ZT 0 under ALF.

### 4.3. Blood Tests

The GLUCOCARD G+ meter was used to measure the serum glucose content (Arkray, Kyoto, Japan). The Rebis Insulin-Mouse T ELISA Kit (Shibayagi, Gunma, Japan) was used to measure the serum insulin levels.

### 4.4. Statistical Analysis

All results are presented as the mean ± standard error of the mean, except for the JTK-Cycle analysis. The daily fluctuations in the individual feeding groups were analyzed using one-way ANOVA (Table 1) followed by the post-hoc Tukey’s multiple comparisons test. Additionally, JTK-Cycle software [51] was used to assess whether the genes displayed daily rhythmic expression (Table 2). The acrophase and *P*-value were obtained by fitting the data on a fixed curve over a 24 h period. Furthermore, the individual feeding groups were analyzed by two-way ANOVA analysis, for the effect of feeding group (feeding), ZT time (time), and interaction (Table 3).

## Figures and Tables

**Table 1:** Results of one-way ANOVA analyses on mRNA expression. Bold font indicates statistical significance.

Tissue	MBH		Liver		WAT		BAT	
Gene	ALF	RF	ALF	RF	ALF	RF	ALF	RF
*Bmal1*	**<0.001**	**<0.001**	**<0.001**	**<0.001**	**<0.001**	**<0.001**	**<0.001**	**<0.001**
*Per2*	**<0.001**	**<0.001**	**<0.001**	**<0.001**	**<0.001**	**<0.001**	**<0.001**	**<0.001**
*Cry1*	0.085	**<0.001**	**<0.001**	**<0.001**	**<0.001**	**<0.001**	**<0.001**	**<0.001**
*Nr1d1*	**<0.001**	**<0.001**	**<0.001**	**<0.001**	**<0.001**	**<0.001**	**<0.001**	**<0.001**
*Npgl*	**0.003**	**0.020**						
*Npgm*	**<0.001**	**0.021**						
*Npy*	**<0.001**	**0.001**						
*Pomc*	0.187	0.166						
*Mch*	**0.045**	0.483						
*Orx*	0.063	0.083						
*Acc*			**<0.001**	**<0.001**	**0.004**	0.899	0.299	**0.004**
*Fasn*			**0.010**	**<0.001**	**<0.001**	0.080	0.268	**0.001**
*Scd1*			**<0.001**	**<0.001**	**0.049**	0.657	0.072	0.208
*Atgl*			**<0.001**	**<0.001**	0.089	0.280	0.125	**<0.001**
*Cpt1a*			**<0.001**	**<0.001**	**<0.001**	0.229	0.434	**<0.001**
*Hsl*			**<0.001**	**<0.001**	**0.004**	**0.002**	**0.043**	0.058

**Table 2:** Results of JTK-Cycle analyses on rhythmicity of mRNA expression. Bold font indicates statistical significance.

Tissue	Gene	*p*-Value		Amplitude		Acrophase	
		ALF	RF	ALF	RF	ALF	RF
MBH	*Bmal1*	**<0.001**	**<0.001**	0.157	0.117	0	12
	*Per2*	**<0.001**	**<0.001**	0.391	0.285	15	7.5
	*Cry1*	**0.021**	**<0.001**	0.095	0.093	16.5	10.5
	*Nr1d1*	**<0.001**	**<0.001**	0.309	0.184	9	1.5
	*Npgl*	0.935	0.268	0.079	0.306	15	3
	*Npgm*	**0.005**	**0.029**	0.278	0.151	19.5	1.5
	*Npy*	1.000	0.338	0.046	0.041	21	7.5
	*Pomc*	1.000	**0.002**	0.106	0.166	12	15
	*Mch*	**0.013**	0.244	0.094	0.061	19.5	9
	*Orx*	0.241	0.098	0.237	0.167	18	12
Liver	*Bmal1*	**<0.001**	**<0.001**	0.520	0.732	22.5	15
	*Per2*	**<0.001**	**<0.001**	7.619	6.659	13.5	4.5
	*Cry1*	**<0.001**	**<0.001**	0.460	0.313	19.5	10.5
	*Nr1d1*	**<0.001**	**<0.001**	8.220	8.185	7.5	22.5
	*Acc*	**<0.001**	**<0.001**	0.797	0.852	13.5	4.5
	*Fasn*	**<0.001**	**<0.001**	0.859	1.212	15	7.5
	*Scd1*	**0.019**	**<0.001**	0.305	0.381	10.5	22.5
	*Atgl*	**<0.001**	**<0.001**	0.853	1.293	9	0
	*Cpt1a*	**<0.001**	**<0.001**	0.802	1.178	9	0
	*Hsl*	**<0.001**	**<0.001**	0.348	0.760	13.5	1.5
WAT	*Bmal1*	**<0.001**	**<0.001**	0.449	0.771	0	12
	*Per2*	**<0.001**	**<0.001**	2.582	1.594	13.5	6
	*Cry1*	**<0.001**	**<0.001**	0.550	0.820	18	10.5
	*Nr1d1*	**<0.001**	**<0.001**	1.727	1.765	7.5	22.5
	*Acc*	**0.005**	1.000	0.174	0.294	18	15
	*Fasn*	**<0.001**	0.072	0.448	0.833	19.5	12
	*Scd1*	0.052	0.946	0.128	0.202	18	1.5
	*Atgl*	**0.014**	**0.011**	0.217	0.202	13.5	1.5
	*Cpt1a*	**<0.001**	0.668	0.299	0.049	7.5	18
	*Hsl*	**<0.001**	**<0.001**	0.306	0.495	12	0
BAT	*Bmal1*	**<0.001**	**<0.001**	0.409	0.582	0	13.5
	*Per2*	**<0.001**	0.053	3.981	0.713	15	6
	*Cry1*	**<0.001**	**<0.001**	0.374	0.648	18	13.5
	*Nr1d1*	**<0.001**	**<0.001**	2.477	1.037	7.5	21
	*Acc*	1.000	**<0.001**	0.083	0.910	6	12
	*Fasn*	1.000	**<0.001**	0.122	3.861	21	12
	*Scd1*	0.209	0.485	0.066	1.205	18	15
	*Atgl*	**0.016**	1.000	0.094	0.049	10.5	0
	*Cpt1a*	**0.017**	**<0.001**	0.105	0.234	9	3
	*Hsl*	**0.008**	0.570	0.136	0.087	12	19.5

**Table 3:** Results of two-way ANOVA analyses on mRNA expression. Bold font indicates statistical significance.

Tissue	Gene	Feeding		Time		Interaction	
MBH	*Bmal1*	F(1,70) = 31.85	**<0.001**	F(7,70) = 1.17	0.333	F(7,70) = 10.62	**<0.001**
	*Per2*	F(1,70) = 0.08	0.779	F(7,70) = 13.20	**<0.001**	F(7,70) = 12.18	**<0.001**
	*Cry1*	F(1,70) = 0.56	0.473	F(7,70) = 6.54	**<0.001**	F(7,70) = 8.67	**<0.001**
	*Nr1d1*	F(1,70) = 1.20	0.299	F(7,70) = 8.42	**<0.001**	F(7,70) = 10.52	**<0.001**
	*Npgl*	F(1,63) = 203.32	**<0.001**	F(7,63) = 2.59	**0.021**	F(7,63) = 2.56	**0.022**
	*Npgm*	F(1,56) = 9.68	**0.014**	F(7,56) = 5.45	**<0.001**	F(7,56) = 2.71	**0.017**
	*Npy*	F(1,63) = 0.83	0.386	F(7,63) = 5.91	**<0.001**	F(7,63) = 2.98	**0.009**
	*Pomc*	F(1,35) = 0.18	0.691	F(7,35) = 1.55	0.185	F(7,35) = 2.04	0.077
	*Mch*	F(1,63) = 0.88	0.372	F(7,63) = 0.93	0.488	F(7,63) = 2.72	**0.016**
	*Orx*	F(1,63) = 1.94	0.197	F(7,63) = 4.33	**<0.001**	F(7,63) = 2.21	**0.045**
Liver	*Bmal1*	F(1,70) = 96.04	**<0.001**	F(7,70) = 37.49	**<0.001**	F(7,70) = 120.42	**<0.001**
	*Per2*	F(1,70) = 3.58	0.088	F(7,70) = 19.92	**<0.001**	F(7,70) = 110.12	**<0.001**
	*Cry1*	F(1,70) = 0.31	0.589	F(7,70) = 17.13	**<0.001**	F(7,70) = 45.10	**<0.001**
	*Nr1d1*	F(1,70) = 4.99	**0.049**	F(7,70) = 42.84	**<0.001**	F(7,70) = 142.87	**<0.001**
	*Acc*	F(1,70) = 1.40	0.263	F(7,70) = 9.79	**<0.001**	F(7,70) = 24.55	**<0.001**
	*Fasn*	F(1,70) = 3.82	0.079	F(7,70) = 9.34	**<0.001**	F(7,70) = 14.25	**<0.001**
	*Scd1*	F(1,70) = 2.46	0.148	F(7,70) = 2.98	**0.009**	F(7,70) = 13.96	**<0.001**
	*Atgl*	F(1,70) = 10.18	**0.010**	F(7,70) = 27.59	**<0.001**	F(7,70) = 92.00	**<0.001**
	*Cpt1a*	F(1,70) = 1.49	0.251	F(7,70) = 26.36	**<0.001**	F(7,70) = 53.11	**<0.001**
	*Hsl*	F(1,70) = 2.86	0.122	F(7,70) = 7.95	**<0.001**	F(7,70) = 33.51	**<0.001**
WAT	*Bmal1*	F(1,70) = 209.59	**<0.001**	F(7,70) = 22.07	**<0.001**	F(7,70) = 117.58	**<0.001**
	*Per2*	F(1,70) = 19.61	**0.001**	F(7,70) = 15.23	**<0.001**	F(7,70) = 49.22	**<0.001**
	*Cry1*	F(1,70) = 0.12	0.734	F(7,70) = 22.43	**<0.001**	F(7,70) = 39.12	**<0.001**
	*Nr1d1*	F(1,70) = 0.55	0.476	F(7,70) = 6.28	**<0.001**	F(7,70) = 47.65	**<0.001**
	*Acc*	F(1,70) = 389.30	**<0.001**	F(7,70) = 0.59	0.761	F(7,70) = 0.38	0.912
	*Fasn*	F(1,70) = 215.27	**<0.001**	F(7,70) = 1.98	0.070	F(7,70) = 2.17	**0.048**
	*Scd1*	F(1,63) = 923.63	**<0.001**	F(7,63) = 1.73	0.119	F(7,63) = 2.04	0.063
	*Atgl*	F(1,70) = 41.25	**<0.001**	F(7,70) = 0.66	0.705	F(7,70) = 2.23	**0.041**
	*Cpt1a*	F(1,70) = 22.35	**<0.001**	F(7,70) = 2.52	**0.023**	F(7,70) = 5.59	**<0.001**
	*Hsl*	F(1,70) = 0.37	0.556	F(7,70) = 0.65	0.710	F(7,70) = 7.33	**<0.001**
BAT	*Bmal1*	F(1,70) = 58.84	**<0.001**	F(7,70) = 8.29	**<0.001**	F(7,70) = 87.98	**<0.001**
	*Per2*	F(1,63) = 20.23	**0.001**	F(7,63) = 49.13	**<0.001**	F(7,63) = 56.73	**<0.001**
	*Cry1*	F(1,63) = 23.80	**<0.001**	F(7,63) = 22.92	**<0.001**	F(7,63) = 25.43	**<0.001**
	*Nr1d1*	F(1,70) = 1.22	0.295	F(7,70) = 10.25	**<0.001**	F(7,70) = 49.35	**<0.001**
	*Acc*	F(1,70) = 1549.45	**<0.001**	F(7,70) = 3.20	**0.005**	F(7,70) = 3.05	**0.007**
	*Fasn*	F(1,70) = 399.79	**<0.001**	F(7,70) = 4.25	**<0.001**	F(7,70) = 4.13	**<0.001**
	*Scd1*	F(1,70) = 509.92	**<0.001**	F(7,70) = 1.38	0.226	F(7,70) = 1.36	0.235
	*Atgl*	F(1,70) = 52.20	**<0.001**	F(7,70) = 3.50	**0.003**	F(7,70) = 2.21	**0.044**
	*Cpt1a*	F(1,70) = 4.10	0.070	F(7,70) = 2.21	**0.044**	F(7,70) = 2.55	**0.021**
	*Hsl*	F(1,70) = 11.44	**0.007**	F(7,70) = 2.96	**0.009**	F(7,70) = 2.65	**0.017**

**Table 4:** Sequences of oligonucleotide primers for quantitative RT-PCR.

Gene	Sense Primer (5’ to 3’)	Antisense Primer (5’ to 3’)
*Bmal1*	ACATAGGACACCTCGCAGAA	AACCATCGACTTCGTAGCGT
*Per2*	TCTGACATGGCTTCTGTTCG	TGTACAGTGTGGGGGTGCTA
*Cry1*	GGGACAGCCAGCTGATGTAT	CATCTCGTTCCTTCCCAAAA
*Nr1d1*	AGCCACCCCAAGACCTTACT	CGGTCATTCAAACTGGACCT
*Npgl*	GGAACCATGGCTTAGGAAGG	TCTAAGGAGCTGAGAATATGCA
*Npgm*	CTCTCTGACGCTGATAGACC	AGATACTGTAATGCCCAGGA
*Npy*	TATCTCTGCTCGTGTGTTTG	GATTGATGTAGTGTCGCAGA
*Pomc*	AGCTGCCTTTCCGCGACA	ATCTATGGAGGTCTGAAGCA
*Mch*	GGAAGGAGAGATTTTGACATGCTC	TTCTTCTGTAAGGATGTTGCGGAC
*Orx*	GCCTCCTTCAGGCCAACGGTAA	GGGGTGCTAAAGCGGTGGTAGT
*Acc*	TCCGCACTGACTGTAACCACAT	TGCTCCGCACAGATTCTTCA
*Fasn*	AGGGGTCGACCTGGTCCTCA	GCCATGCCCAGAGGGTGGTT
*Scd1*	CTGTACGGGATCATACTGGTTC	GCCGTGCCTTGTAAGTTCTG
*Atgl*	AACACCAGCATCCAGTTCAA	GGTTCAGTAGGCCATTCCTC
*Cpt1a*	CCTGGGCATGATTGCAAAG	GGACGCCACTCACGATGTT
*Hsl*	GCTGGGCTGTCAAGCACTGT	GTAACTGGGTAGGCTGCCAT
*Actb*	GGCACCACACCTTCTACAAT	AGGTCTCAAACATGATCTGG
*Rps18*	CCTGAGAAGTTCCAGCACAT	TTCTCCAGCCCTCTTGGTG

## Data Availability

No big data repositories needed. The raw data supporting the findings of this manuscript will be made available by the corresponding author, K.U., to any qualified researcher upon reasonable request.

## Supplementary Materials

The following are available online at https://www.mdpi.com/1422-0067/22/4/2109/s1.

## Abbreviations

ACC	Acetyl-CoA carboxylase
ACTB	Beta-actin
ALF	Ad libitum feeding
ATGL	Adipose triglyceride lipase
BAT	Brown adipose tissue
BMAL1	Brain and muscle ARNT-like 1
CPT1a	Carnitine palmitoyl transferase 1a
CRY1	Cryptochrome 1
DMH	Dorsomedial hypothalamus
FAA	Food anticipatory activity
FASN	Fatty acid synthase
HSL	Hormone-sensitive lipase
MBH	Mediobasal hypothalamus
MCH	Melanin-concentrating hormone
NPGL	Neurosecretory protein GL
NPGM	Neurosecretory protein GM
NPY	Neuropeptide Y
NR1D1	Nuclear receptor subfamily 1 group D member 1
ORX	Orexin
PER2	Period 2
POMC	Proopiomelanocortin
RPS18	Ribosomal protein S18
SCD1	Stearoyl-CoA desaturase 1
SCN	Suprachiasmatic nucleus
RF	Time-restricted feeding
WAT	White adipose tissue
ZT	Zeitgeber time
