# Supplementary material for: Effects of Irregular Feeding on the Daily Fluctuations in mRNA Expression of the Neurosecretory Protein GL and Neurosecretory Protein GM Genes in the Mouse Hypothalamus
Source: Int J Mol Sci. 2021 Feb 20;22(4):2109. doi: 10.3390/ijms22042109 (PMC7924315; doi:10.3390/ijms22042109)
Supplement: Supplementary file 1 [file ijms-22-02109-s001.pdf]

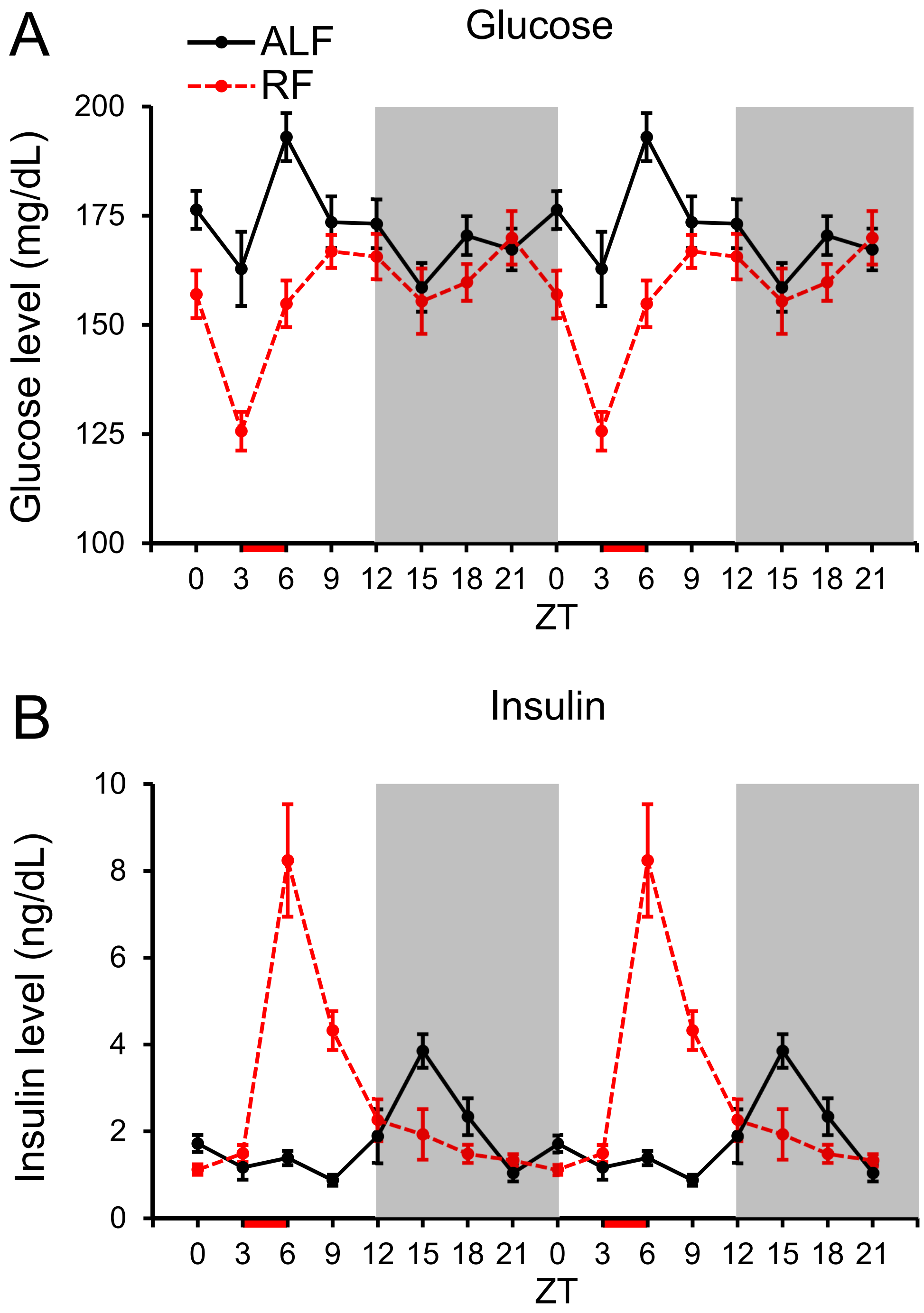

Figure S1. Daily profiles of the blood serum glucose (**A**) and insulin levels (**B**) in mice under ad libitum feeding (ALF: Black lines) and time-restricted feeding (RF: Red lines) schedules. Data are expressed as the mean ± standard error of the mean, n = 5–6 mice per datum point.

Table S1. Results of one-way ANOVA analyses on blood glucose and insulin levels.

	ALF	RF
Glucose	**0.021**	**<0.001**
Insulin	**<0.001**	**<0.001**
